# Allergic diseases in the elderly

**DOI:** 10.1186/2045-7022-1-11

**Published:** 2011-10-17

**Authors:** Victoria Cardona, Mar Guilarte, Olga Luengo, Moises Labrador-Horrillo, Anna Sala-Cunill, Teresa Garriga

**Affiliations:** 1 Allergy Section, Department of Internal Medicine, Hospital Universitari Vall d'Hebron, Barcelona, Spain; 2 Allergy Research Unit, Institut de Recerca Vall d'Hebron, Universitat Autònoma de Barcelona, Spain

**Keywords:** Allergy, asthma, elderly, aging population, immunosenescence, drug allergy, pharmacotherapy, dermatitis

## Abstract

Demographic distribution of the population is progressively changing with the proportion of elderly persons increasing in most societies. This entails that there is a need to evaluate the impact of common diseases, such as asthma and other allergic conditions, in this age segment. Frailty, comorbidities and polymedication are some of the factors that condition management in geriatric patients. The objective of this review is to highlight the characteristics of allergic diseases in older age groups, from the influence of immunosenescence, to particular clinical implications and management issues, such as drug interactions or age-related side effects.

## Introduction

People 65 years old or more are the fastest growing segment of the population in the developed countries. By 2030, it is estimated that this group will comprise about 20% of the total population, and among elderly persons, the percentage of patients aged above 80 years will increase disproportionately. The prevalence of allergic diseases, in the elderly is estimated around 5-10% [1,2]. Although allergic conditions are often thought of as childhood disorders, the disease often persists into older age and can occasionally make its initial appearance in the elderly.

Specific issues that arise when investigating allergies in the elderly patients are several. First of all, the definition of older persons needs to be clarified in order to homogenize nomenclature when addressing this entity. Usually, the term older adults is applied to persons 65 years or older, since it takes into account not only the chronologic aspect of aging, but also the fact that around this is retirement age in many countries. Subclassification into several ranges after this age may take into account increasing frailty, comorbidities and dependence.

A number of factors in older subjects contribute to their risk for developing allergic related conditions. These include frailty, coexisting medical problems, memory issues and use of multiple prescribed and non prescribed medications [3]. However, more studies should be designed so as to know the prevalence and particularities of allergy in the elderly since data in this field are scarce. Also, recruitment of older subjects into clinical trials is necessary to provide a reliable evidence base to facilitate the identification of safe and effective diagnostic and therapeutic methods for elderly patients with suspected allergic conditions.

### Underlying mechanisms of allergic diseases in the elderly

#### Immunosensecence

The complex process of immunosenescence, the aging of the immune system, affects both the innate and the adaptive immune system. The clinical consequences include not only increased susceptibility to infection, malignancy and autoimmunity, but also decreased response to vaccination, and impaired wound healing. The most severe clinical impact is probably a result of the loss of diversity in the T-cell-receptor and B-cell-receptor repertoire, owing to the accumulation of dysfunctional cells, and decreased thymic and bone-marrow output. Immunosenescence in the innate immune system appears to reflect dysregulation, rather than exclusively impaired function. Changes of inmunosenescence features on immune cells are summarized in Table 1[4-7].

**Table 1:** Summary of immunosenescence features

Cell type	Changes with aging
Neutrophils	Reduced phagocytosis
	Reduced reactive oxygen species production
	Defect in apoptotic cell death

Eosinophils	Reduced degranulation
	Reduced superoxide production

Mast cells	Reduced degranulation
	Dysregulations in function

Monocytes/macrophages	Reduced phagocytosis
	Reduced cytokine and chemokine secretion
	Reduced generation of nitric oxide and superoxide

Dendritic cells	Reduced phagocytosis and pinocytosis
	Increased IL-6 and TNF-alfa production
	Diminished TLR expression and function
	Dysregulations in function

T cells	Reduced response and proliferation
	Reduced CD28 expression
	Reduced TCR diversity
	Reduced signal transduction
	Dysregulations in function

B cells	Production of low-affinity antibodies
	Increased oligoclonal expansion
	Decline in serum total IgE values
	Reduced surface MHC class II molecule expression
	Dysregulations in function

Epitelial cells	Impaired production of cytokines
	Decreased clearance of particles

NK cells and NKT cells	Reduced numbers or increased in several tissues Reduced cytotoxicity and proliferation

Adapted from [4-7]

Some molecules such as zinc, vitamin D or iron, seem to play a relevant role in maintaining the immune response. Data reported clearly suggest that the essential trace element, zinc (Zn) plays a pivotal role for the immune efficiency. It is required to reach healthy aging and longevity. The intracellular zinc homeostasis is regulated by buffering metallothioneins (MT) and zinc transporters (ZnT and ZIP families) that mediate the intracellular zinc signalling assigning to zinc a role of "second messenger". In aging, the intracellular zinc homeostasis is altered, because high MT are unable to release zinc and some zinc transporters deputed to zinc influx (ZIP family) are defective leading to low intracellular zinc content for the immune efficiency. Physiological zinc supplementation in the elderly improves these functions in some cases, although it remains unclear which old subjects who effectively need zinc supplementation [8].

Serum concentrations of vitamin D are generally lowered in older compared with younger subjects [9]. Calcitriol, the active form of vitamin D, influences innate and adaptive immunity. It acts on APC and T cells to promote peripheral tolerance via inhibition of inflammatory responses and induction of regulatory T cells [10]. Epidemiologic studies highlight the increasing prevalence of vitamin D deficiency and insufficiency and its association with an increased risk of autoimmune diseases and poor respiratory function, including asthma. There seems to be a non-linear association between calcitriol and IgE concentrations [11].

Clinical observations supported by epidemiological data, have demonstrated age-related declines in serum total immunoglobulin E (IgE) and in allergen sensitization in the elderly. Serum total IgE comparisons between younger and older subjects without any allergic disease have demonstrated significantly lower levels in the older subjects [12]. Nevertheless, atopic disorders are complex diseases that involve interactions among several physiological systems, skin, lung, mucosae, and the immune system and are also present in the elderly and seems that immunosenescence does not affect increased IgE levels in atopic patients with AD and/or high serum IgE levels indicating that in these subgroups of patients the atopic propensity remains into advanced age [13]. In spite of these observations, skin tests and specific IgE to diagnose inhalant, food or drug allergy can and should be used both in younger and older patients [14-16].

#### Specific organ changes

Typical symptoms of allergic rhinitis like nasal obstruction, postnasal drip or cough may be worsened by the anatomic and physiological changes of the nose that occur with age [17]. Structural changes, such as retraction of the nasal columella due to weakening of septal cartilage and a loss of nasal tip support, may decrease nasal airflow leading to exacerbation of nasal obstruction, complaints commonly seen in geriatric rhinitis patients. Postnasal drip and cough are common AR symptoms that can be worsened by thickened mucus along with decreased mucociliary clearance with age. Temperature and humidity values in the nasal cavity are significantly lower in the geriatric population, which could explain the nasal irritation symptoms related to dryness and crusting [18]. Moreover, it has recently been described that the severity of symptoms of allergic rhinitis are significantly correlated to the mucociliary transport time, which slows with age [19].

Although asthma is nowadays considered a heterogeneous disease with many phenotypes and endotypes accounting for differential clinical expression, several biologic processes related to aging seem to be involved in the physiopathology of asthma in the elderly. Genetic, epigenetic and environmental factors are known to account for these changes. Processes such as cell aging, progressive loss of lung function during life, together with anomalies in the inflammation associated with asthma, may be some of the hallmarks of asthma in this age group. Current knowledge and further needs in research have recently been reviewed in a workshop of the National Institute on Aging (NIA) of the United States [20].

Skin aging is characterized by atrophy of the epidermis and dermis due to loss of hydration, as a result of chronologic and environmental factors [21]. Characteristic of aging is a progressive loss of function and structural integrity resulting in impaired immune response and skin barrier function, vascular impairment, metabolic imbalance of reactive oxygen species, and components of the extracellular matrix [22].

Elderly patients may develop allergic contact dermatitis (ACD) despite the fact that cell-mediated immunity is decreased as a result of unknown mechanisms. Absolute T and B cell counts are not reduced with age. Age does not seem to affect the number of Langherhans cells in the elderly skin compared with the nonelderly skin [23]. It has been reported that both CD4+ and CD8+ subsets probably contribute to the immune response in ACD because they both have the capacity to produce IL-17 and are present in significant numbers in the infiltrate [24].

Murine as well as human studies indicate that physiological changes affecting also the local immune responses might contribute to the development of food allergy. The induction of mucosal tolerance has been shown to be impaired in aged animals, while the effector phase is maintained [25], so oral tolerance established in young age is usually maintained in senescence while the ingestion of new dietary proteins may induce *de novo *sensitization. One of the first-line gastrointestinal defence mechanisms against ingested antigens is secretory IgA. It has been reported that orally induced antigen-specific IgA responses are weakened in aged animals [26]. Iron deficiency, a prevalent condition in geriatric populations, can also have a role in the development of food allergy in older patients. A reduction of iron leads to a decrease of IgG4 responses [27], known to prevent activation of effector cells by capturing the allergen before it can cross-link cell-bound IgE. An impairment of intestinal permeability may also predispose to the development of food allergy. Both animal [28] and human studies [29] have shown an increase of intestinal permeability with aging. Extrinsic factors which may increase permeability such as alcohol [30,31], or which induce hypoacidic conditions such as proton pump inhibitors [32] or antiacid drugs[33] have also been related to increased food allergy.

### Clinical features of allergic diseases in the elderly

#### Allergic rhinitis

Although the peak incidence is during adulthood, allergic rhinitis (AR) is prevalent among older people affecting around 5.4 to 10.7% of patients above 65 years [31,34,35]. Patients typically present with sneezing, pruritus and nasal congestion. It has been described that patients over 50 years and older have a higher frequency of isolated ocular symptoms [36]. A detailed allergy history is crucial in the diagnosis of AR in older individuals, because this population is at particular risk for other types of rhinitis that may cause similar symptoms [37]. Reduction of blood flow to the nose can lead to progressive nasal atrophy and clinical manifestations of atrophic rhinitis, and thinning and dryness of the mucosa make the elderly especially prone to rhinitis medicamentosa.

#### Asthma

Asthma affects older patients just as in any other age group. The prevalence of asthma in the elderly in developed societies is estimated around 6-10%. Fatalities are higher in older asthmatics compared to other age groups in which mortality is steadily decreasing [38].

Diagnosis of asthma is hampered by certain limitations in elderly asthmatics, rendering an estimate that only half of asthmatics patients being diagnosed [39]. Symptoms characteristic of asthma, such as wheeze, cough, chest tightness or dyspnoea, may not be as straight forward in this age group and are easily mistaken as expression of other common conditions such as bronchitis, chronic obstructive pulmonary disease (COPD) or heart failure. Also, it seems clear that objective diagnostic tests such as spirometry, bronchodilator response or non-specific bronchial challenge tests are underused in elderly patients [40,41]. Nevertheless, it has been shown that spirometry can be adequately performed by the majority of older patients [42,43]. In some cases, when previous tests are not confirmatory, a course of oral steroids may aid in establishing if bronchial obstruction is reversible or not [44]. Further diagnostic tests will be needed in order to rule out other conditions or comorbidities such as parenchymal lung disease, COPD, gastro-oesophageal reflux, sleep apnoea or congestive heart failure (Table 2). Assessment of inhalant sensitisation has been shown to be useful in aged patients to classify their asthma and to consider etiologic measures such as avoidance or even specific immunotherapy in selected cases [16].

**Table 2:** Assessment and management issues of asthma in older patients

	Assessment
Comorbidities	

COPD	Smoking history, spirometry, 6-min walking test, oxygen saturation,...

Congestive heart failure	Chest X-ray, echocardiogram, B-type natriuretic peptide

Gastro-oesophageal reflux	Proton pump inhibitor trial, oesophageal pHmetry

Sleep apnoea	Sleep monitoring

Anaemia	Haemoglobin

Mood and mental disorders	Psychological evaluation, specific test scores for depression, anxiety, dementia

Risk factors	

Smoking	Self report, exhaled carbon monoxide

Excessive or deficient body weight	Body mass index

Activity limitation	Self reported

Management	

Inhaler device technique	Direct assessment

Exacerbation management	Self report

Polymedication	Medication review

Common conditions affected by asthma drugs	

Cataracts	Ophthalmic examination

Osteoporosis	Densitometry

Hyperglycemia	Glycaemia monitoring

Adapted from Gibson PG et al. [38]

#### Cutaneous allergic conditions

Clinically, aged skin is characterized by atrophy, wrinkling, fragility, alterations in pigmentation, a higher frequency of benign and malignant tumours, and a greater tendency to xerosis. Altogether, these factors contribute to greater susceptibility to dermatologic diseases and pruritus, the most common skin complaint in patients over the age of 65 years, frequently associated with dry skin [45-47].

Systemic pruritus is defined as pruritus arising from disease of organs other than the skin, such as liver, kidney, or blood. This type of pruritus should be differentiated from neurologic pruritus, which can arise from neuronal damage (neuropathic itch) or can have neurogenic origin without neuronal damage, such as itching after administration of opioids (neurogenic itch). Systemic diseases, including also some neurologic disorders that may be responsible for pruritus, are mentioned in Table 3[48]. Psychogenic pruritus, also called somatoform pruritus, constitutes a distinct category and may accompany various psychiatric conditions [49]. Many systemic and topical drugs can induce pruritus, so this adverse effect should be taken into account when assessing old patients with chronic itch [50].

**Table 3:** Systemic diseases accompanied by generalized pruritus/urticaria

Liver diseases	Primary biliary cirrhosis
	Primary sclerosing cholangitis
	Extrahepatic cholestasis
	Heptatits B and C
Kidney diseases	Chronic kidney insufficiency

Hematologic diseases	Polycythemia vera
	Hodgkin disease
	Non-Hodgkin lymphomas
	Leukemias
	Myeloma Multiplex
	Iron deficiency
	Systemic mastocytosis
	Hypereosinophilic syndrome
	Myelodysplastic syndromes

Endocrine disorders	Diabetes
	Hyperthyroidism
	Hypothyroidism
	Hyperparathyroidism

Neurologic diseases (neuropathic pruritus)	Brain injury/tumor (frequently unilateral pruritus)
	Sclerosis multiplex
	Small fiber neuropathy

Solid tumors (paraneoplasic pruritus)	

Carcinoid syndrome	

Infectious diseases	HIV infection/AIDS
	Infestations

Adapted from Reich A et al. [48]

ACD in the elderly is not uncommon [24,51,52]. The most common contact allergens are nickel sulphate (11-12%) and fragrance/balsam of Peru (7-9%) [24,53]. Topical medications, such as those used for leg ulcers, are a common cause of ACD [24,52,54]. Neomycin and the corticosteroids, often give late positive reactions; it is therefore important to perform delayed readings of patch tests [55,56]. Also, patients who have undergone ostomies for gastrointestinal or urinary disorders often develop irritant or allergic stomal dermatitis. Allergens involved include adhesives, fragrances, rubber chemicals and acrylates [57]. Photopatch testing should be carried out whenever there is a photodistributed eczematous rash [55].

Atopic dermatitis is much less common in the elderly compared with children and young adults. It is associated with seasonal mucosal allergies, asthma and positive prick tests to various allergens. Late onset atopic dermatitis, without the usual history of atopy, may explain eczema of unknown origin and negative patch tests [58]. Scabies should enter the differential diagnosis in generalised dermatitis, as institutional acquisition of scabies is common in the elderly.

Urticaria (with or without angioedema), especially chronic spontaneous urticaria, is quite common in elderly patients. Systemic diseases that may induce urticaria should be ruled out (table 3). Angioedema in the absence of urticaria can be due to overproduction of bradykinin. It is exceptional that hereditary deficiency in the C1 inhibitor (HAE-C1-INH) has its onset in the elderly. On the other hand, it is more frequent that acquired C1 inhibitor deficiency (AAE-C1-INH) presents in the older age [59], and it is characterized by massive activation of the classical complement pathway and accelerated catabolism of C1-INH due to lymphatic tissue neoplasms or autoimmune diseases. Although there are no published data of the prevalence of this condition, it seems to be low. In contrast, the prevalence of angiotensin-converting enzyme inhibitor angioedema (AE-ACEi) is quite high, ranging from 0.1%-2.2% [60,61] and it should be suspected in all patients with AE who are receiving ACEi. Normal levels of complement factors help to reinforce clinical suspicion and to rule out the possibility of AE with C1-INH deficiency.

#### Food allergy

Although epidemiological studies reveal an increase in food allergies (FA) in industrialized countries these are mainly focused on children and young adults. Nevertheless, a proportion of patients will face a persistence of their problem. Also, previously unaffected individuals may develop symptoms of food allergy during adulthood for the first time. In the Allergy Section at Hospital Vall d'Hebron, we have observed a prevalence of 5% of FA in our outpatients older than 65 years, compared to 26% in patients aged 40 to 65 and 69% in younger patients (16-39 years old) (data not published). The profile of sensitization to different foodstuffs does not differ among groups except for *Rosaceae *fruits and fish, which are more frequent in young patients. Regarding clinical manifestations, anaphylaxis is less common in older patients [62].

#### Drug allergy

Adverse drug reactions (ADR) are very common in frail elderly, cause clinically significant morbidity and mortality and are associated with large economic costs [63-66]. ADR are observed 2 to 3 times more frequently in geriatric patients than in adult patients younger than 30 years. It is estimated that ADR are responsible for up to 10% of hospital admissions in older patients with overdosing being a significant problem in the elderly [67-69].

Drugs most commonly implicated in type I, II and III hypersensitivity reactions in older patients include beta-lactam drugs, nonsteroidal anti-inflammatory drugs (NSAIDS), quinolones, trimethoprim-sulfamethozaxole, nitrofurantoin, heparin, neuromuscular blocking agents, antiepileptics, chemotherapeutic agents, monoclonal antibodies and immunosuppressive drugs [70-74]. On the other hand, systemic drugs most commonly implicated in causing type IV reactions are antiepileptics, allopurinol, dapsone, abacavir and nevirapine [75].

The evaluation of a potential drug allergy in the elderly may include a thorough history, skin tests (prick and/or intradermal test, or patch tests) and in selected cases oral, intramuscular or intravenous challenge tests. However, it is important to evaluate the extent of comorbidity and functional impairment that is present in elderly patients prior to the initiation of a drug allergy study. For this reason, a comprehensive geriatric assessment (CGA) can be particularly useful [76]. Most outpatient CGA programs exclude patients who are unlikely to benefit of some therapeutic plans because of terminal illness, severe dementia and complete functional dependence or inevitable nursing home placement [77,78].

Polymedication is a limitation in drug allergy assessment. Older ambulatory patients use approximately three times as many medications as younger patients. Hence, the use of large numbers of medications by older patients increases the likelihood of harmful drug interactions, adverse drug reactions and drug allergies and, in occasions, makes it difficult for the allergist to identify the suspected medication responsible for the ADR [79].

### Pharmacology in allergic elderly patients

The potential for drug interaction increases with age and with the number of drugs prescribed [80]. The most important mechanisms for drug-drug interactions are the inhibition or induction of drug metabolism, and pharmacodynamic potentiation or antagonism. This is because the elderly have reduced homeostatic mechanisms, decreased renal function and a biotransformation in the liver may also play a role and are therefore particularly sensitive to, for example, the combined postural hypotensive or sedative effects of drugs [81]. Strategies to avoid drug-drug interactions in the elderly include an appropriate alerting system in computers in general practice, exercising care in prescribing, monitoring patients regularly, paying special attention to institutionalised and frail elderly patients, auditing drug interactions and reporting drug interactions to the regulatory authorities.

When prescribing a treatment for AR in the elderly, the possibility of drug-drug interactions, and the impact of drug treatment on concomitant diseases should be taken into account. Nasal steroids, topical antihistamines and non-sedating antihistamines are particularly suited for management of AR in the elderly both for safety and efficacy. Clearance of leukotriene receptor antagonists is decreased in the elderly, and has the potential to interact with a wide range of drugs that inhibit or induce the CYP 3A4 or 2C9 systems [34]. As a general rule, first-generation antihistamines should not be used for AR in the elderly due to risks of side effects and interactions with other medications [82]. Topical and systemic decongestants should also be avoided because they may aggravate nasal dryness and cause systemic side effects such as confusion, difficulty in urination, irritability and aggravation of glaucoma. Non-pharmacologic treatment should include nasal lavage with isotonic sodium chloride for reducing nasal dryness and clearing thick mucus [83].

Management of asthma in older patients seems suboptimal, and the use of inhaled corticosteroids is low, although they are probably the best maintenance therapy in most patients [37,84]. Nevertheless, medical evidence in this age group is very limited due to the systematic exclusions of elderly people, smokers or patients with concomitant COPD from trials, and subsequently guidelines derived from such evidence may not be fully applicable to older patients or those with several comorbidities. In the case of older asthmatics, all these issues should be carefully assessed when establishing management, as well as possible drug interactions, capability to use inhaler devices and patient preferences.

The use of long-acting β-adrenergic agonists (LABAs) has a synergic effect with inhaled corticosteroids. Although older patients with coronary disease may be more prone to adverse side effects, they are generally considered safe, but discontinuation should be considered when control of asthma is achieved [85,86]. Also short-acting β-adrenergic agonists can cause cardiotoxicity in case of overdosing. In some cases, the use of an anticholinergic aerosol could be a therapeutic option. Other alternative treatments such as leukotriene receptor antagonists, which may be useful in some aspirin sensitive patients, or theophylline, may be considered. Nevertheless, due to its narrow therapeutic window, careful monitoring is advised if theophylline is used. Influenza and pneumococcus immunization protect against these respiratory infections which are directly related to a significant number of asthma exacerbations, and should therefore be recommended in older asthmatics [87].

Options for treatment of skin allergic disorders include topical and systemic drugs. Topical treatments may prevent major adverse effects. Cooling agents such as menthol, may decrease the intensity of itching by activation of low temperature receptors in the skin. Anaesthetics with formulations based on benzocaine or lidocaine are widely used, especially in neuropathic pruritus, but they can induce ACD and may induce side effects in the circulatory system [88]. Antihistamines such as topical doxepine are effective in atopic and ACD. Other topical antihistamines may induce contact allergy [89]. Capsaicin owes its antipruritic properties to desensitization of sensory nerve fibres and it has shown to be effective in nostalgia paraesthetica, prurigo nodularis and uremic pruritus [88]. Topical corticosteroids have limited value in the treatment of pruritus; they might only be effective in inflammatory skin disorders. Calcineurin inhibitors are potent antipruritic drugs in patients with atopic dermatitis [90]. N-palmitoylethanolamine, a cannabinoid receptor CB2 agonist, is a promising new compound that activates cannabinoid receptors in the skin and has shown to reduce pruritus in atopic dermatitis, lichen simplex, prurigo nodularis, and chronic kidney disease-associated itching [91].

Systemic treatment options for pruritus include sedating antihistamines, antidepressants, μ- or κ-opioid receptor agonists and neuroleptics. Most of these drugs are not devoid of relevant side effects, such as drowsiness. Therefore it is usually recommended to start at low doses in the elderly and to taper up [92]. UVB phototherapy is effective especially in uremic pruritus, cholestasic pruritus and HIV-associated pruritus [93]. Psychotherapy is helpful in the treatment of somatoform pruritus or neurotic excoriations.

Atopic dermatitis, and all of the others forms of dermatitis that are recalcitrant to other therapies, can be treated with topical or systemic corticosteroids. If they are ineffective or adverse effects preclude the use of corticosteroids, treatment with calcineurin inhibitors, phototherapy, or systemic immunosuppressives such as cyclosporine could be used [55].

#### Antihistamines

First-generation H1 receptor antagonists are lypophilic and therefore may cross the blood-brain barrier. Elderly persons may be at greater risk of adverse effects involving the CNS, such as confusion, sedation, dizziness, sleepiness or impaired cognitive function [94]. It has been shown that diphenhydramine administration in older hospitalized patients over 70 years of age is associated with increased risk of cognitive decline compared with nonexposed patients [95]. Because of the lack of specificity for the H1 receptor, first generation antihistamines also have additional dopaminergic, serotoninergic, muscarinic and cholinergic adverse effects [96]. Thus, particularly in the elderly, there is a higher risk of urinary hesitancy, urinary retention, constipation, as well as arrhythmias, peripheral vasodilatation, postural hypotension or tachycardia. These side effects may lead to falls or aggravation of concomitant diseases such as prostatic hypertrophy, glaucoma and heart disease. In a recent study on the inappropriate drug use and mortality in community-dwelling elderly, first-generation antihistamines accounted for one of most frequent drugs prescribed although contraindicated [97]. In the light of these findings, first-generation antihistamines should be prescribed with extreme caution in elderly patients.

Second-generation antihistamines have a lower capacity to cause CNS-related adverse effects as they have a low potential to cross the blood-brain barrier, and provide selective H1 blockade without anticholinergic or alpha-adrenoreceptor antagonist activity. Some second-generation antihistamines are metabolized by the cytochrome 450 enzyme system in their first pass through the liver, which may lead to drug-drug interactions or elevated plasma drug interactions in patients with liver dysfunction and others are excreted through the kidneys and dosage has to be adjusted according renal function [82]. Second-generation oral H1 antihistamines potentially requiring a dose reduction in patients with hepatic dysfunction include cetirizine, ebastine, levocetirizine, and loratadine. Those potentially requiring a dose reduction in patients with renal dysfunction include cetirizine, ebastine, fexofenadine and levocetirizine [98]. According to the studies of Affrime et al in healthy subjects, no dosage adjustment for desloratadine is required in the elderly [99].

#### Corticosteroids

Topical and oral corticosteroids are particularly useful in the treatment of acute and delayed hypersensitivity diseases. However, they have adverse effects on many organ system, and these range from those that are not necessarily serious (e.g. cushingoid appearance), to those that are life-threatening (e.g. serious infections)[100].

Some of these adverse effects may be aggravated in the elderly. Patients receiving prednisolone 5-40 mg/day for at least 1 year have a partial loss of explicit memory, and elderly patients may be more susceptible to memory impairment with less protracted treatment [101].

The risk of developing diabetes mellitus is increased by more than two-fold in elderly patients who are newly initiated on oral corticosteroid therapy [102]. An increased risk for peptic ulcer disease has been reported in corticosteroid users who were receiving NSAIDs concurrently, persons receiving NSAIDs and corticosteroids have a risk for peptic ulcer disease that is 15 times greater than that of nonusers of either drug [103]. This finding is especially important in allergy practice because patients receiving oral corticosteroids are likely to be receiving NSAIDs as well, given that aspirin or other NSAIDs are among the most prescribed drugs in old age [100].

Other frequent conditions of old age such as cataracts [104] or osteoporosis [105] have also been related to the use of systemic but also of high doses of inhaled corticosteroids, and should be borne in mind when treating allergic geriatric patients.

### Specific immunotherapy in the elderly

Altered function of aged immune system is primarily associated with exposure to "new" antigenic challenges, as is the case for decreased efficient therapeutic value of vaccinations among the elderly population. While influenza vaccination has shown to reduce influenza-related mortality, current influenza vaccines have limited efficacy in elderly (17-53%) compared to vaccine efficacy in young adults (70-90%). To provide better protective measures for the increasingly aging population new and improved vaccines are needed. Currently, a variety of approaches are being explored to enhance the efficacy of influenza vaccines in the elderly, including the use of adjuvants, altering antigen dose and identifying the best routes of vaccine administration [106]. There is not similar evidence with specific immunotherapy. There are few studies of use of this treatment in older adults.

Specific immunotherapy (SIT) is deemed the only treatment that can at least partly modify the natural course of the disease during its initial stages. Its use in elderly patients is still debated.

Injection SIT can be considered an effective therapeutic option in otherwise healthy elderly patients with a short disease duration whose symptoms cannot be adequately controlled by drug therapies alone [107,108]. One study also describes that sublingual immunotherapy (SLIT) reduces symptoms, drug consumption and the progression of the disease in both young and elderly subjects allergic to house-dust mites, with persistent rhinitis and mild bronchial asthma [109].

## Conclusions

Allergic diseases do affect elderly patients and special considerations regarding diagnosis and treatment should be taken into account when assessing these patients. Clinical manifestations may be less straightforward than in younger age groups, hampering the recognition of the disease, and rendering a more complicated differential diagnosis. Treatment options may be limited by comorbidities and ongoing medication use, which may give rise to deleterious effects on the allergic disease or to drug interactions with anti-allergic medications. Therefore, further research is warranted in this area.

## Abbreviations

AAE-C1-INH: acquired C1 inhibitor deficiency; ACD: allergic contact dermatitis; ADR: adverse drug reactions; AE: angioedema; AE-ACEi: angiotensin-converting enzyme inhibitor angioedema; AR: allergic rhinitis; CGA: comprehensive geriatric assessment; COPD: chronic obstructive pulmonary disease; FA: food allergies; HAE-C1-INH: hereditary deficiency in the C1 inhibitor; IgE: Immunoglobulin E; LABAs: long-acting β-adrenergic agonists; MT: metallothioneins; NK cells: natural killer cells, NKT cells: natural killer T cells; NKT cells: natural killer T cells; NSAIDS: nonsteroidal anti-inflammatory drugs; SIT: specific immunotherapy; TCR: T cell receptor; TLR: toll like receptors; Zn: zinc.

## Competing interests

The authors declare that they have no competing interests.

## Authors' contributions

Each author was responsible to draft a part of the manuscript. All authors read and approved the final manuscript.
